# Medium Roasting and Brewing Methods Differentially Modulate Global Metabolites, Lipids, Biogenic Amines, Minerals, and Antioxidant Capacity of Hawai‘i-Grown Coffee (*Coffea arabica*)

**DOI:** 10.3390/metabo13030412

**Published:** 2023-03-10

**Authors:** Pratibha V. Nerurkar, Jennifer Yokoyama, Kramer Ichimura, Shannon Kutscher, Jamie Wong, Harry C. Bittenbender, Youping Deng

**Affiliations:** 1 Laboratory of Metabolic Disorders and Alternative Medicine, Department of Molecular Biosciences and Bioengineering (MBBE), College of Tropical Agriculture and Human Resources (CTAHR), University of Hawai‘i at Manoa (UHM), Honolulu, HI 96822, USA; 2 Department of Tropical Plant and Soil Sciences (TPSS), CTAHR, UHM, Honolulu, HI 96822, USA; 3 Bioinformatics Core, Departmentt of Quantitative Health Sciences, University of Hawai‘i Cancer Center (UHCC), John A. Burns School of Medicine (JABSOM), UHM, Honolulu, HI 96813, USA

**Keywords:** coffee, *Coffea arabica*, brewing, roasting, metabolomics, lipidomics, biogenic amines

## Abstract

In the United States, besides the US territory Puerto Rico, Hawai‘i is the only state that grows commercial coffee. In Hawai’i, coffee is the second most valuable agricultural commodity. Health benefits associated with moderate coffee consumption, including its antioxidant capacity, have been correlated to its bioactive components. Post-harvest techniques, coffee variety, degree of roasting, and brewing methods significantly impact the metabolites, lipids, minerals, and/or antioxidant capacity of brewed coffees. The goal of our study was to understand the impact of roasting and brewing methods on metabolites, lipids, biogenic amines, minerals, and antioxidant capacity of two Hawai‘i-grown coffee (*Coffea arabica*) varieties, “Kona Typica” and “Yellow Catuai”. Our results indicated that both roasting and coffee variety significantly modulated several metabolites, lipids, and biogenic amines of the coffee brews. Furthermore, regardless of coffee variety, the antioxidant capacity of roasted coffee brews was higher in cold brews. Similarly, total minerals were higher in “Kona Typica” cold brews followed by “Yellow Catuai” cold brews. Hawai‘i-grown coffees are considered “specialty coffees” since they are grown in unique volcanic soils and tropical microclimates with unique flavors. Our studies indicate that both Hawai‘i-grown coffees contain several health-promoting components. However, future studies are warranted to compare Hawai‘i-grown coffees with other popular brand coffees and their health benefits in vivo.

## 1. Introduction

Coffee is one of the most popular drinks worldwide and is a widely consumed beverage. During 2022–2023, global coffee consumption is expected to reach over 168.7 million 60 kg (132 lb.) bags [1], and it is forecasted that the global production will be 172.8 million bags in the 2022–2023 growing season [2]. In the United States, it is estimated that more than 157 million cups of coffee are consumed per day, making the United States the world’s leading coffee consumer [3]. The health benefits of coffee, including its antioxidant properties, are associated with its complex array of bioactive chemicals, the most influential being alkaloids (caffeine and trigonelline), phenolic compounds (chlorogenic acids), and diterpenes (cafestol and kahweol). Epidemiological studies have reported several health benefits of coffee such as reducing the risk of metabolic syndrome (MetS), type 2 diabetes (T2D), cardiovascular disease (CVD), various types of cancer, kidney stones, liver disease, Parkinson’s disease, gout, and neurological disorders [4,5,6,7]. Moderate coffee consumption may also confer protective effects against overall mortality [8,9,10,11,12,13]. However, a few studies indicate that drinking more than four to nine cups of coffee per day may have some detrimental health effects such as increasing plasma cholesterol or triglyceride levels, and decreasing bone density in women [6,14,15,16]. Consuming large amounts of caffeinated coffee can also have negative effects on health such as increased blood pressure, anxiety, or difficulty falling asleep [17]. Abrupt cessation of caffeine consumption may induce withdrawal symptoms such as headache, fatigue, and/or depression. Coffee consumption during pregnancy is associated with adverse birth outcomes and neonatal health including lower infant birth weight [18,19,20,21,22,23,24,25,26,27,28]. Similarly, biogenic amines identified in coffee brews, resulting from amino acid decarboxylation, can be toxic to humans at high concentrations [29]. Inconsistencies observed in the health benefits of coffee may possibly be associated with differences in the chemical compositions of coffee [30,31,32,33,34,35,36].

It has been demonstrated that differences in coffee metabolites and caffeine levels [36,37] are influenced by their geographic origins or environment [38,39,40,41,42,43,44], post-harvest processing [30,33,45,46,47,48], instant versus fresh ground coffees [49,50], degrees of roasting [33,51,52,53], and/or types of brewing methods [31,33,36,49,54,55,56]. Different coffee metabolites not only impart flavors [38,57,58] or aromas [59,60,61,62,63], but also determine therapeutic impact by influencing antioxidant potential [34,64,65,66,67]. Prospective epidemiological studies conducted among populations of Hawai‘i indicate that a moderate level of coffee consumption (one to three cups per day) was associated with a lower risk of T2D, chronic liver diseases, dementia, and Parkinson’s disease [68,69,70,71]. In contrast, drinking more than nine cups of coffee per day was positively associated with increased serum cholesterol among several population-based studies, including Japanese men in Hawai‘i [14,15].

Besides the US territory Puerto Rico, Hawai‘i is the only state in the United States that grows commercial coffee. In Hawai’i, coffee is the second most valuable agriculture commodity after seed crops [72,73]. In 2021, unroasted coffee was valued at USD 102.91 million, and its roasted value was more than USD 148.48 million [74]. In 2022, annual revenue for the US coffee industry was estimated at USD 90.27 billion [75]. Hawai’i-grown coffees account for less than 1% of total global coffee production. However, they are considered specialty coffees since they are grown in unique volcanic soils and tropical microclimates with unique flavors [76]. The main varieties of coffee grown in Hawai‘i are “Kona Typica” and “Yellow Catuai”, which represent two major *Coffea arabica* botanical varieties of “Typica” and “Bourbon” used worldwide.

Metabolomic profiles of coffees brewed by different methods have been reported [33,36]. However, studies on the effect of roasting conditions on coffee metabolites are limited [33]. The antioxidant capacities and mineral contents of several coffee varieties and the impact of roasting conditions have also been reported [64,77,78,79,80,81,82,83]. Green coffee beans (grounds) are generally not brewed for consumption. Only roasted coffee grounds are brewed for consumption worldwide. Roasting generates the flavor of coffee. Green coffee brews would not be considered “coffee” since they do not taste like the “coffee” brewed from roasted grounds. Overall, no studies have addressed the lipidomic profiles of brewed coffee from roasted beans, while only one study evaluated selected biogenic amines in brewed coffee [29]. To date, one study has identified metabolites from spent grounds of Hawai‘ian Kona coffee [84]. However, variability in metabolites, lipids, biogenic amines, mineral contents, or antioxidant capacity of Hawai‘i-grown coffee (*Coffea arabica*) has yet to be elucidated.

The primary objective of our study was to identify the impact of roasting and brewing methods on global metabolites, lipids, and biogenic amines in the two Hawai‘i-grown coffee varieties, “Kona Typica” and “Yellow Catuai”. Drip filter paper, drip filter mesh and French press are the most widely used methods for brewing roasted coffee. For our initial investigation, to evaluate the differences in metabolites, lipids, and biogenic amines among brews of roasted and green coffee beans, we used the above three brewing methods of coffee preparation. However, to understand the effects of different methods of brewing roasted coffee, we also included the cold brewing method, which is gaining popularity worldwide and has been reported to provide more beneficial compounds than other brewing methods [85,86]. The secondary objective of our study was to evaluate the influence of brewing methods on the antioxidant capacity and mineral contents of these two Hawai‘i-grown coffees. Brewing green coffee bean grounds is also not common. Therefore, we analyzed the minerals and the antioxidant capacity of brews prepared only from roasted coffee grounds.

## 2. Materials and Methods

Green coffee beans, 50 lbs. each, of “Kona Typica” (purchased from Waialua Coffee and Cacao Estate, Oahu, Honolulu, HI, USA) and “Yellow Catuai” (purchased from Kaua’i Coffee Company, Kaua’i, Kalaheo, HI, USA) were stored at room temperature. “Kona Typica” is also grown in Kaua’i, and “Yellow Catuai” is grown in Kona and on Oahu. Although metabolite differences are noted based on genetic variety, geographic location can also make a difference [38,39,40,41,42,43,44,46]. For ease of readership and future study reference, we will refer to “Kona Typica” as Waialua and “Yellow Catuai” as Kaua’i coffee.

### 2.1. Green Coffee Grounds

Nylon bags filled with green coffee were dunked in liquid nitrogen for three to five seconds in order to freeze the green coffee beans. The frozen, dried green coffee beans were ground using a “Santos burr grinder” (Sao Paulo, Brazil). Grounds were collected in a jar, and larger grounds that did not pass completely through were discarded. Green coffee grounds were brewed using the drip filter paper, drip filter mesh and French press methods described below.

### 2.2. Coffee Roasting

Prime-grade green beans were further cleaned by hand to remove any defective green beans and roasted using the electric, programmable rotating drum type “Has Garanti roaster” (Model HSR 1 kg (92.2 lbs.), Turkey), at 230 °C temperature for about 13 min, until coffee reached a medium roast color according to the “Roast Color Classification System” (Agtron 55-65, SCAA, Long Beach, CA, USA), and cooled at room temperature. Coffee beans were ground using the “Santos coffee grinder” (Burr type, Santa Fe Springs, CA, USA) to a size for paper filter brewing. For each type of brewing method, three roasted batches were mixed thoroughly and pooled as one sample. To understand inter-roasting variability, a total of six batches were roasted, providing two pooled samples for preparing the different brews.

### 2.3. Drip Filter Paper (FP) Method

Two tablespoons (Tbsp) of coffee grounds with six fluid ounces (fl oz) of room-temperature tap water was brewed in the “Toastmaster” coffee machine (Star International Holdings group of brands, Star manufacturing, Smithville, TN, USA) using the “Total Home #4 Cone Style paper Filter” (CVS Pharmacy, Honolulu, HI, USA). After brewing was completed, the hot plate was turned off, and the coffee was cooled in the pot for 15 min. Cooled coffee brews were aliquoted into 50 mL tubes and stored at −80 °C until lyophilization.

### 2.4. Drip Metallic Filter Mesh (FM) Method

Six fl oz of room-temperature tap water with two Tbsp of coffee grounds was brewed in the “Black and Decker Brew ‘N Go” (San Diego, CA, USA) coffee machine, which contains a metallic (steel) mesh filter. After brewing was completed, coffee was cooled in “Brew ‘N Go cup” for 15 min, aliquoted into 50 mL tubes, and stored at −80 °C until lyophilization.

### 2.5. French Press (FrP) Method

Six fl oz of boiling water with two Tbsp of coffee grounds was set to steep for 15 min in the “Bodum Brazil 3 cup French Press Coffee Maker 12 oz” (Bodum incorporation, Triengen, Switzerland). The ground coffee beans were pressed with a perforated plunger plate and then cooled for 20 min in the brewing container. The coffee brew was then aliquoted into 50 mL tubes and stored at −80 °C until lyophilization.

### 2.6. Cold Brew (CB) Method

A damp, reusable “Toddy Filter” (fabric-like, compostable filter made from tree-free specialized material by Toddy, LLC (Loveland, CO, USA) ) was placed in the bottom of the “Toddy Cold Brew System” (Toddy, LLC, Loveland, CO, USA). One fl oz of room-temperature tap water and 4 Tbsp of roasted coffee grounds were added to the system. An additional three fl oz of water was gently added to the mixture and left to sit for 5 min. Another batch of four Tbsp of grounds and three fl oz of water was carefully added. The grounds were lightly pressed down to ensure all grounds were wet. The system was covered with foil and kept to steep for 24 h. After steeping, the coffee was measured and 2 times the amount of water was added to dilute the concentrate. Ten-milliliter aliquots were distributed, frozen, and stored in a −80 °C freezer until lyophilization. Samples were lyophilized in a Martin Christ Alpha 2-4 LD plus (Christ, Osterode am Harz, Germany) for 24 h, pooled, and then stored in a −80 °C freezer until analysis.

### 2.7. Brewed Coffee Omics (Global Metabolites, Lipids, and Biogenic Amines)

Roasted coffee grounds were brewed by drip filter paper, drip filter mesh, French press, and cold brew methods. Green coffee bean grounds were brewed by drip filter paper, drip filter mesh, and French press methods. Metabolomics, lipidomics, and analysis of biogenic amines were conducted at the Fiehn Laboratory, NIH West Coast Metabolomics Center. Global metabolites (targeted and untargeted) were analyzed using an automated liner exchange cold injection system gas chromatography time of flight mass spectrometer (ALEX-CIS GCTOF MS) as described previously [87,88,89,90,91]. In brief, 10 mg brewed coffee samples were extracted with 1 mL of 3:3:2 acetonitrile (ACN):isopropanol (IPA):water by vortexing for 10 s and shaking for 6 min at 4 °C. After centrifugation at 14,000 RCF (relative centrifugal force) for 2 min, the supernatant was aliquoted into 475 μL aliquots, dried, and stored until further analysis. Half of the dried sample was derivatized with 10 μL of 40 mg·mL^−1^ of methoxyamine in pyridine and shaken at 30 °C for 1.5 h. Ninety-one microliters of N-methyl-N-(trimethylsilyl) trifluoroacetamide (MSTFA) + fatty acid methyl esters (FAMEs) was added to each sample and further shaken at 37 °C for 0.5 h to complete derivatization. Then, 0.5 μL derivatized samples were injected on a 7890A gas chromatogram (GC) coupled with a time of flight mass spectrometer (TOF; LECO Corporation, St. Joseph, MI, USA) using a splitless method onto a RESTEK RTX-5SIL MS column with an Intergra-Guard at 275 °C with a helium flow of 1 mL.min^−1^. The GC oven was set at 50 °C for 1 min and then ramped to 330 °C at the rate of 20 °C.min^−1^ and held for 5 min. The transfer line was set to 280 °C and the EI ion source was set to 250 °C. The MS parameters collect data from 85 m/z to 500 m/z at an acquisition rate of 17 spectra/s.

Lipids were determined using the charged surface hybrid column electrospray method using a quadrupole time of flight mass spectrometer and tandem mass spectrometry (CSH-ESI QTOF MS/MS) as described previously [92,93]. In brief, 10 mg brewed coffee samples were vortexed with LCMS grade methanol (225 μL) and methyl tert-butyl ether (MTBE, 750 μL) and extracted by shaking for 6 min at 4 °C as previously described [90,93]. Samples were then vortexed with LCMS-grade water (188 μL). After centrifugation at 14,000 RCF (relative centrifugal force) for 2 min, the polar and non-polar layers were separated, dried, and stored until further analysis. Free fatty acids (FFAs); mono-, di-, and triglycerides (TGs); cholesteryl ester (CE); phospholipids (PLs); and sphingolipids (SLs) were analyzed by CSH-ESI QTOF MS/MS. Data were collected in both positive electrospray ionization (ESI) and negative ESI mode. Peaks were annotated by comparing MS/MS spectra and accurate masses of the precursor ion to spectra provided in the Fiehn Laboratory’s LipidBlast spectral library [94,95]. To profile biogenic amines, samples were analyzed using the hydrophilic interaction chromatography electrospray method, using a quadrupole time of flight mass spectrometer and tandem mass spectrometry (HILIC-ESI QTOF MS/MS) as described previously [90,93]. Data were collected in both positive and negative ESI modes and processed using MS-DIAL and MS-FLO programs as described [96].

### 2.8. Mineral and Metal Analysis of Brewed Coffee

The brewed coffees were lyophilized and sent to the Agricultural Diagnostic Service Center (ADSC), College of Tropical Agriculture and Human Resources (CTAHR), for analysis. The minerals analyzed included boron (B), calcium (Ca), copper (Cu), iron (Fe), magnesium (Mg), manganese (Mn), molybdenum (Mo), phosphorus (P), potassium (K), sodium (Na), and Zinc (Zn). The metals that were analyzed included arsenic (As), cadmium (Cd), chromium (Cr), nickel (Ni), lead (Pb), selenium (Se), and vanadium (Va). Three independent brews for each type of coffee and each brewing method were analyzed in triplicate.

Minerals and metals were analyzed by the standard EPA 3050B method (https://www.epa.gov/sites/default/files/2015-06/documents/epa-3050b.pdf, accessed on 5 January 2023) with slight modifications. In brief, 0.5 g lyophilized brewed coffee samples were used for analysis. Samples were digested with 3. 5 mL of concentrated nitric acid (15.8 N) for about 10 min at 110 °C. The samples were then mixed with 100 mL of ddH2O, incubated on a shaker, filtered through Whatman 42 filter paper, and analyzed on an Avio 560 Max Inductively Coupled Plasma Optical Emission Spectrometer (ICP-OES, Perkin Elmer, Waltham, MA, USA).

### 2.9. Antioxidant Capacity of Brewed Coffee

The antioxidant activity of brews prepared from roasted coffee grounds was analyzed using a commercial ORAC Antioxidant Assay Kit, Cat# AOX-2-RB(Zen-Bio, Inc., Research Triangle Park, North Carolina). The ORAC is a kinetic assay that measures fluorescein decay and antioxidant protection over time. It is an assay that is regularly used to measure the total antioxidant capacity of biological fluids, cells, and tissues as well as dietary supplements, therapeutics [97], and food extracts [98]. In this assay, the substrate 2,2′-azobis-2-methyl-propanimidamine dihydrochloride (AAPH) generates a peroxyl radical (ROO^-^) which is formed upon thermal homolysis at 37 °C. This peroxyl radical oxidizes fluorescein, which thereby produces a non-fluorescent product. The principle of the assay relies on the hydrogen atom transfer (HAT) mechanism of antioxidants to suppress oxidative degradation of the signal and measures the subsequent fluorescent activity vs. reaction time [99]. ORAC measures the inhibition of the peroxyl radical-induced oxidation as carried out by antioxidant compounds in coffee. The fluorescent measurements were expressed relative to the initial reading (excitation/emission at 485 nm/528–538 nm). Three independent brews for each type of coffee and each brewing method were analyzed in triplicate at four different concentrations of 1:100, 1:200, 1:500, and 1:750. Time course reactions of fluorescein decay from AAPH were measured using a 96-well plate reader (Perkin Elmer Wallac 1420 Victor2) and plate reading software (Wallac 1420).

Raw data from the ORAC assays were exported from the Wallac 1420 software to Excel and were normalized according to the following equation: AUC = 0.5 + (F1/F0) + (F2/F0) + … + 0.5 (F30/F0), where F0 = initial fluorescence reading at time = 0 min and F1, F2, etc., are the fluorescence readings at different time points. The area under the curve (AUC) values were generated using GraphPad Prism 7.0 for Mac OS X, version 7.0e. The net AUC was determined by subtracting the AUC of the blank from that of the coffee sample. A Trolox standard curve was generated using the net AUC vs. µM Trolox, in accordance with the one site-specific binding model (GraphPad Prism 7, GraphPad Software, CA, USA). Trolox equivalencies were determined by the one-site specific equation (Michaelis–Menten), as determined by the following equation: Y= Bmax * X/(Kd + X), where X is the equivalent Trolox concentration for coffee, Y is the net AUC for each coffee dilution, and Vmax and Kd are values from the Trolox standard curve. The ORAC data are represented as Trolox equivalences (TEs) of coffee. Three independent experiments were performed in replicates of six (n = 18).

### 2.10. Statistical Data Analysis

All coffee metabolites, lipids, and biogenic amines (known and unknown) were analyzed by univariate analysis using the parametric *t*-test to compare the mean differences of two groups. The one-way analysis of variance (ANOVA) was followed by Holm–Sidak correction for multiple comparisons. The known metabolites were further subjected to multivariate analysis using MetaboAnalyst 5.0 (https://www.metaboanalyst.ca/docs/Publications.xhtml, accessed between 6 September and 20 October 2022) [31]. Data were normalized to sample median, log_10_ transformed and Pareto scaled. Principal component analysis (PCA) was used to observe clustering trends and exclude outliers. A discriminant model was created by further subjecting the data to partial least squares discriminant analysis (PLS-DA). One thousand permutation tests were performed to check model validity and potential over-fitting of the PLS-DA model and visualized using a validation plot. Known metabolites, lipids, and biogenic amines in each category were used to build the PLS-DA models. After validation of the PLS-DA model, data were further analyzed by orthogonal partial least squares discriminant analysis (OPLS-DA) to identify discriminant metabolites, lipids, and biogenic amines and distinguish between categories at false discovery rate (FDR) < 0.05.

Data for mineral and metal contents and ORAC assays are expressed as a mean ± standard deviation of triplicate or quadruplet values. Statistical significance was assessed using GraphPad Prism 7.0. Data were analyzed using either a two-tailed unpaired *t*-test or one-way ANOVA followed by Tukey’s test. *p* values < 0.05 were considered to be significant.

## 3. Results

A total of 442 metabolites tentatively assigned as global metabolites (139 known and 303 unknown), 1617 metabolites tentatively assigned as positive ESI lipids (80 known and 1537 unknown), 2862 metabolites tentatively assigned as negative ESI lipids (40 known and 2822 unknown), and 1747 metabolites tentatively assigned as biogenic amines (47 known and 1700 unknown) were detected in both coffee varieties.

### 3.1. Differences in Global Metabolites, Lipids, and Biogenic Amines among the Green and Roasted Coffee Varieties of “Kona Typica” (Waialua) and “Yellow Catuai” (Kaua’i) Brews

Univariate analysis indicated that 92 metabolites tentatively assigned as global metabolites (36 known and 54 unknown) were significantly different between the green varieties of Kaua’i and Waialua coffee (*p* < 0.05, supplement file 1). Similarly, 32 metabolites tentatively assigned as lipids (positive ESI; 4 known and 28 unknown), 103 metabolites tentatively assigned as negative ESI lipids (10 known and 93 unknown), and 132 metabolites tentatively assigned as biogenic amines (9 known and 123 unknown) were significantly different between the green varieties of Kaua’i and Waialua coffee (*p* < 0.05, supplement file 1).

**Figure 1:**
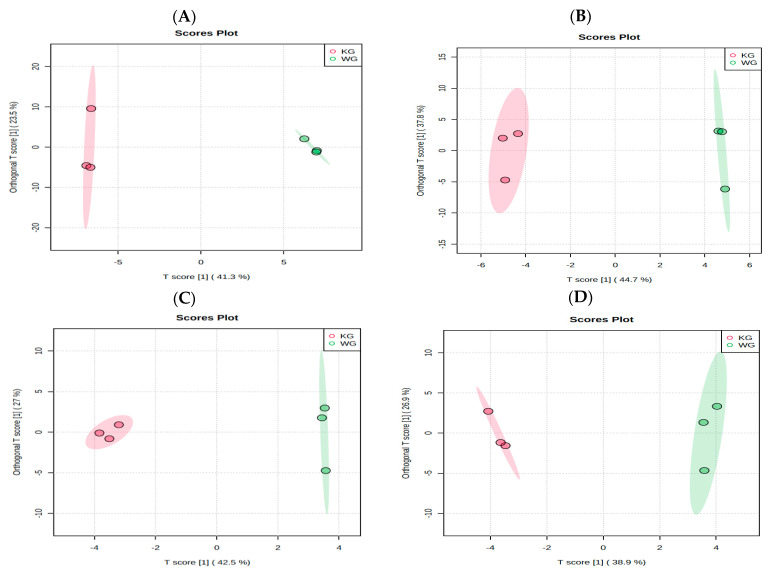
OPLS-DA score plots for brews of Kaua’i green (KG, n = 3) and Waialua green (WG, n = 3) coffees for known (**A**) global metabolites, (**B**) positive ESI mode lipids, (**C**) negative ESI mode lipids, and (**D**) biogenic amines.

Kaua’i green coffee brews contained higher amounts of 2-hydroxybutanoic acid (14.64-fold), LPC (18:0; 37.97-fold), chlorogenic acid (18:34-fold), and N6,N6,N6-Trimethyllysine (12.53-fold) as compared to Waialua green coffee brews (*p* < 0.05, supplement file 1). Orthogonal projections to latent structures discriminant analysis (OPLS-DA) score plots for green varieties of Kaua’i and Waialua coffee are represented in Figure 1 as determined by multivariate analysis.

Brews of the roasted coffees also demonstrated differences in 134 metabolites tentatively assigned as global metabolites (44 known and 89 unknown), 145 metabolites tentatively assigned positive ESI lipids (5 known and 140 unknown), 146 metabolites tentatively assigned as negative ESI lipids (12 known and 134 unknown), and 433 metabolites tentatively assigned as biogenic amines (15 known and 418 unknown) which were significantly different among Kaua’i and Waialua coffees (*p* < 0.05, supplement file 2).

**Figure 2:**
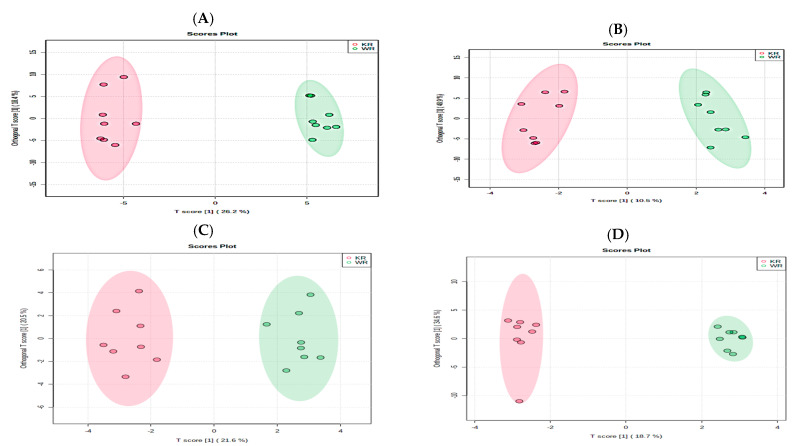
OPLS-DA score plots for brews of roasted Kaua’i coffee (KR, n = 3) and roasted Waialua coffee (WR, n = 3) for known (**A**) global metabolites, (**B**) positive ESI mode lipids, (**C**) negative ESI mode lipids, and (**D**) biogenic amines. 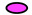 = Kaua’i roasted (KR) and 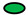 = Waialua roasted (WR).

Among the known compounds, galactinol, pyrogallol, uracil, phosphatidyl choline (PC) (34:1), PC (34:2), PC (36:2), PC (16:0), PE (38:2), PE (36:1), PC (38:2), PC (36:4), PC (36:2), PC (36:1), PC (35:4), PC (34:1), and DL-Indole-3-lactic acid were more than 2-fold higher in roasted Kaua’i coffee brews as compared to the roasted Waialua coffee brews (*p* < 0.05, supplement file 2). The OPLS-DA score plots for roasted Kaua’i and Waialua coffee brews are represented in Figure 2.

### 3.2. Influence of Roasting on Metabolites, Lipids, and Biogenic Amines of “Kona Typica” (Waialua) and “Yellow Catuai” (Kaua’i) Coffee Brews

**Figure 3:**
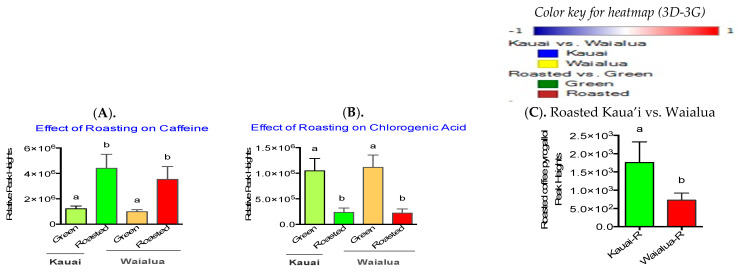

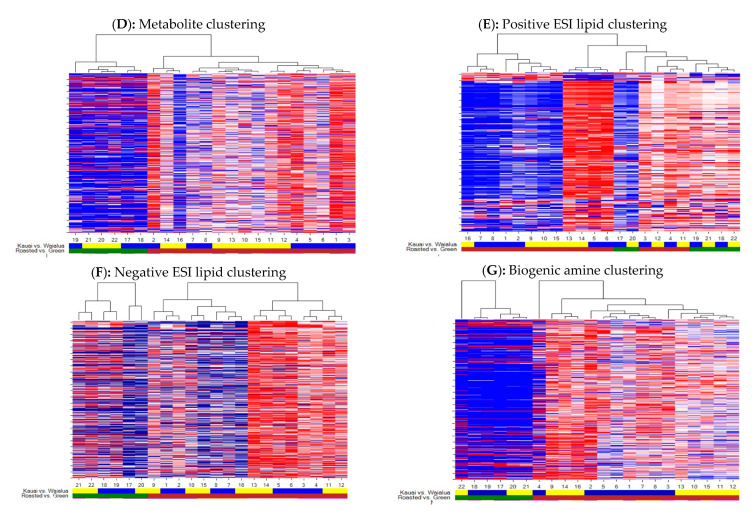
Effect of roasting on caffeine (**A**), chlorogenic acid (**B**), and pyrogallol (**C**), analyzed using GraphPad Prism 7.0. Values are mean ± SD (n = 8). ^a,b^ Mean values with common letters do not differ (*p* < 0.05). Heatmaps of Kaua’i and Waialua coffee for known global metabolites (**D**), positive ESI mode lipids (**E**), negative ESI mode lipids (**F**), and biogenic amines (**G**).

As expected, roasting significantly increased caffeine and reduced chlorogenic acid in both coffee varieties (Figure 3A and Figure 3B, respectively). Similar effects of roasting on caffeine and chlorogenic contents of coffee have been reported by others [100]. Roasted Kaua’i coffee contained significantly high amounts of pyrogallol (Figure 3C, *p* < 0.05) compared to Waialua coffee. Overall differences in Kaua’i and Waialua coffee varieties as well as the effect of medium roasting conditions are noted in Figure 3D–G which depict hierarchal clustering (heatmaps) of all identified and unidentified metabolites for the green and roasted coffee brews: (C) global metabolites, (D) positive ESI lipid profiles, (E) negative ESI lipid profiles, and (G) biogenic amines.

**Figure 4:**
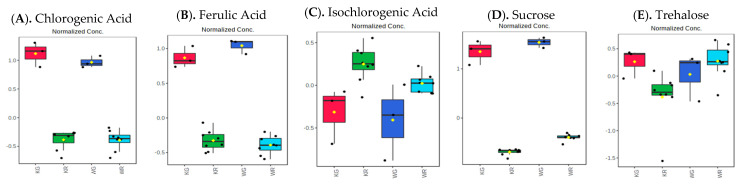

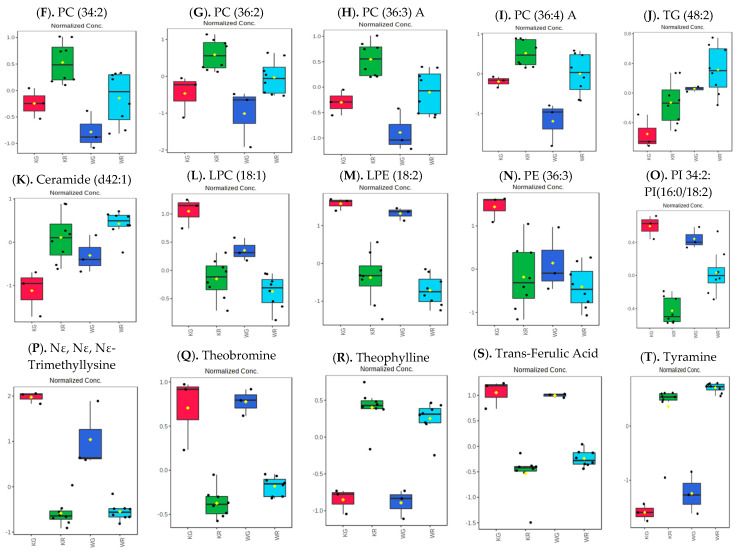
Relative changes of selected known metabolites (**A**–**E**), positive ESI lipids (**F**–**J**), negative ESI lipids (**K**–**O**), and amines (**P**–**T**) in green (*n* = 3) vs. roasted (*n* = 8) coffees (*p* < 0.05). 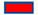 KG = Kaua’i green, 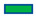 KR = Kaua’i roasted, 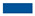 WG = Waialua green, and 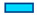 WR = Waialua roasted.

Roasted Kaua’i coffee brews demonstrated significant differences in 102 known and 209 unknown metabolites tentatively assigned as global metabolites, 119 metabolites tentatively assigned as positive ESI mode lipids (5 known and 114 unknown), 678 metabolites tentatively assigned as negative ESI mode lipids (8 known and 670 unknown), and 1132 metabolites tentatively assigned as biogenic amines (38 known and 1094 unknown) as compared to the green Kaua’i coffee brews (*p* < 0.05, supplement file 3). Figure 4 compares relative changes in selected metabolites, lipids, and biogenic amines of green and roasted Kaua’i coffee brews (adjusted *p* value (FDR) < 0.05).

Similarly, 312 metabolites tentatively assigned as global metabolites (103 known and 209 unknown), 105 metabolites tentatively assigned as positive ESI mode lipids (5 known and 100 unknown), 697 metabolites tentatively assigned as negative ESI mode lipids (7 known and 690 unknown), and 1129 metabolites tentatively assigned as biogenic amines (38 known and 1091 unknown) were significantly different in roasted Waialua coffee brews as compared to the green Waialua coffee brews (*p* < 0.05, supplement file 4). Figure 4 depicts comparative selected box plots of known metabolites (Figure 4A–E), positive ESI lipids (Figure 4F–J), negative ESI lipids (Figure 4K–O), and biogenic amines (Figure 4P–T) as analyzed using MetaboAnalyst 5.0 (supplement file 5).

### 3.3. Influence of Brewing Methods on Metabolites, Lipids, and Biogenic Amines of “Kona Typica” (Waialua) and “Yellow Catuai” (Kaua’i) Coffee Brews

A total of six batches were roasted from each coffee variety. Three roasted batches were pooled into one sample, and the procedure was repeated twice to understand inter-roasting variability. Each variety of roasted coffee was brewed twice by four different brewing methods (n = 2 for each method). Known metabolites, lipids, and biogenic amines for both coffee types were compared for each brewing method by volcano plots using GraphPad Prism 7.0. For example, the cold brew of roasted Kaua’i coffee was compared with the cold brew of roasted Waialua coffee. Unpaired data were analyzed using parametric *t*-tests. The threshold for p value comparison was set at *p* < 0.05 and corrected for multiple comparisons using the Holm–Sidak method. No significant differences were noted among both coffee varieties brewed by the same brewing method. Therefore, data from both coffee varieties were combined for each brewing method (n = 4) and were analyzed using MetaboAnalyst 5.0.

**Figure 5:**
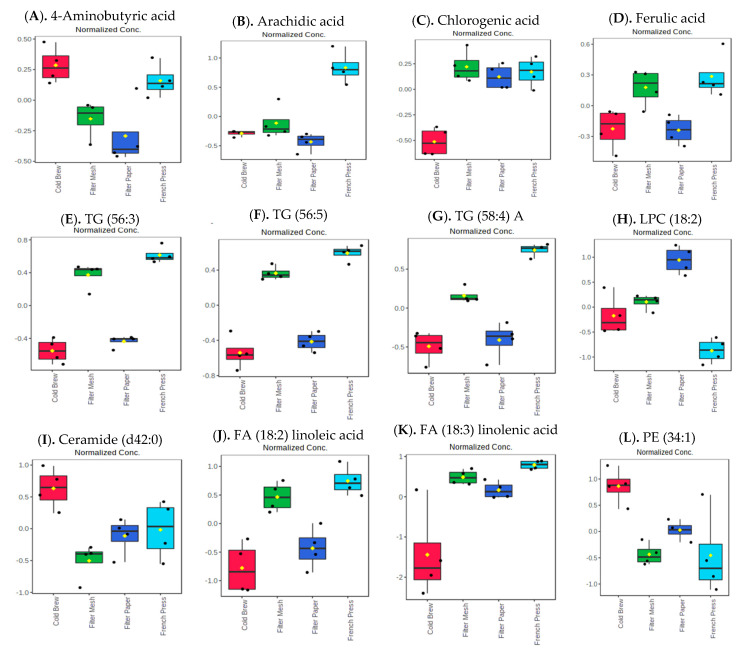
Relative changes of selected known metabolites (**A**–**D**) and positive (**F**–**H**) and negative (**I**–**L**) ESI mode lipids in different coffee brews are depicted (*n* = 4, *p* < 0.05). TG, triacylglycerol; LPC, lysophosphatidylcholine; FA, fatty acid; PE, phosphatidylethanolamine. 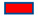 cold brew; 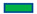 filter mesh; 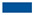 filter paper; 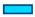 French press.

Twenty-two metabolites tentatively assigned as global metabolites (11 known and 11 unknown) were significantly different in the four types of coffee brews as determined by one-way ANOVA followed by Fisher’s least significant difference method (Fisher’s LSD, adjusted p value (FDR) *p* < 0.05; supplement file 6). Interestingly, brewing methods significantly affected levels of several lipids. About 891 (49 known and 842 unknown) metabolites tentatively assigned as positive ESI mode lipids and 1447 (12 known and 1435 unknown) metabolites tentatively assigned as negative ESI mode lipids were significantly different in each type of brew (*p* < 0.05, supplement file 6). Selected significant metabolites (Figure 5A–D), positive ESI mode lipids (Figure 5E–H), and negative ESI mode lipids (Figure 5I–L) are depicted in Figure 5 below. Among the metabolites tentatively assigned as biogenic amines, only nine unknown amines were significantly different in the four types of brews (*p* < 0.05, supplement file 6).

### 3.4. Mineral and Metal Analysis of “Kona Typica” (Waialua) and “Yellow Catuai” (Kaua’i) Coffee Brews

Brews of roasted coffee grounds are most widely consumed worldwide as compared to brews of green beans. Hence, the mineral and metal profiles of only the roasted coffee bean brews were analyzed. As indicated in Table 1, the amounts of most minerals in cold brews were significantly higher compared to those brewed by the French press, filter paper, and filter mesh brewing methods (Table 1, *p* < 0.05). Although not statistically significant, total minerals were higher in W-CB > K-CB > K-FrP > W-FrP > W-FM > K-FM > W-FP > K-FP (Table 1).

An 8 oz cup of cold brew Kaua’i coffee contained 5.19%, 13.73%, 1.66%, 13.79%, 1.79%, 0.44%, 22.85%, 0.45%, and 3.15% of the RDA for P, K, Ca, Mg, Na, Fe, Mn, Zn, and Cu, respectively. All RDA values were calculated based on recommended values (National Academy of Sciences, 2019). The amounts of minerals in cold brew Kaua’i coffee were in the order of K > Mg > P > Na > Ca > Mn > B > Fe > Zn > Cu. For all other brews of Kaua’i coffee, the order of mineral abundance was K > Mg > Na > P > Ca > Mn > B > Fe > Zn = Cu. In general, except for boron, Kaua’i coffee brewed by filter paper method had the lowest mineral contents, followed by filter mesh < French press < cold brew.

Mineral contents in brewed Waialua coffee also showed similar trends to those observed in Kaua’i coffee (Table 1). An 8 oz cup of cold brew Waialua coffee contained 6.03%, 17.16%, 1.99%, 15.29%, 1.99%, 0.75%, 37.05%, 0.74%, and 11.83% of the RDAs for P, K, Ca, Mg, Na, Fe, Mn, Zn, and Cu, respectively. For Waialua coffee, most of the mineral contents were similar in those brewed by filter paper and filter mesh except for K, while the amounts of minerals in French press coffee were less than those in cold brews.

The Food and Nutrition Board has not established an RDA or AI for boron, which also does not have a DV. Total median boron intakes from dietary supplements and foods are about 1.0 to 1.5 mg.day^−1^ for adults (NIH.gov, accessed on 10 November 2022).

### 3.5. Antioxidant Capacity of “Kona Typica” (Waialua) and “Yellow Catuai” (Kaua’i) Coffee Brews

**Figure 6:**
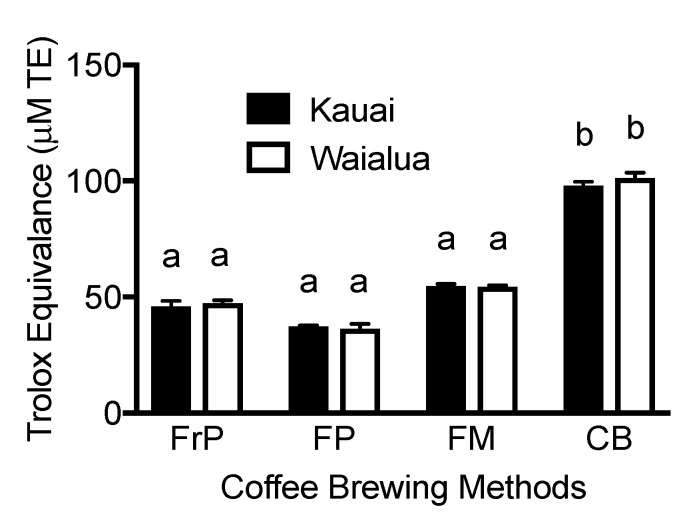
Antioxidant capacity of Kaua’i and Waialua coffee. FrP, French press; FP, filter paper; FM, filter mesh; CB, cold brew; K, Kailua; W, Waialua. Data are represented as mean ± SD (n = 18). ^a,b^ Mean values with common letters do not differ (*p* < 0.05).

The antioxidant capacity, as measured by ORAC values, was influenced by the methods of brewing rather than the coffee variety. The highest ORAC values were noted for the cold brewing method and were about 2.6- to 2.8-fold higher compared to the filter paper method (Figure 6, *p* < 0.05). For both coffee varieties, the antioxidant capacities for different brewing methods were in the order of cold brew > filter mesh > French press > filter paper (Figure 6). Overall, there were no significant differences between the ORAC values for Kaua’i and Waialua coffee brews prepared by the same brewing method (Figure 6, *p* < 0.05).

## 4. Discussion

To understand the effects of health benefits and/or disease risks of coffee consumption, several studies have identified the metabolite composition of commercially brewed coffees based on the brewing methods, roast levels, coffee varieties, and/or caffeine contents [31,33,36,49,51,52,53,54,55,56]. The goal of our study was to identify differences in metabolites, lipids, and biogenic amines between the two types of Hawai‘i-grown coffees and the effects of roasting and brewing methods. Previous studies have used coffee beans to identify varietal differences in metabolites, while we used coffee brews [41,101]. The metabolomic approach has been previously used to identify specialty coffees and characterize their quality and exotic coffee tastes. For example, higher levels of sucrose and lower levels of γ-aminobutyric acid (GABA), quinic acid, choline, acetic acid, and fatty acids were observed in specialty or high-grade green coffees [102]. Similarly, arachidic acid and stearic acid were identified as markers for the Bourbon genealogical group; myristic and linoleic acids and sucrose, for the exotic genotype coffees; and lauric, palmitoleic, and oleic acids, for the Timor Hybrid group [103]. Metabolomic profiling has been also used to identify differences in Philippine coffee to distinguish between *Coffea arabica* (Arabica) and *Coffea canephora* var. Robusta coffees [41], sensory values [104], geographic diversity of Indonesian arabica coffees [105], defective coffee seeds of Brazilian coffee [101], and degrees of coffee adulteration in civet coffee blends [39]; discriminate Arabica and Robusta blends [106,107,108]; compare caffeinated and decaffeinated coffee [37]; and compare fermented coffees [109]. In keeping with published studies, the two Hawai‘i-grown coffee varieties also demonstrate distinct profiles of several metabolites in green beans as well as roasted beans. As previously noted by others [33], roasting significantly reduced chlorogenic acid and increased caffeine in both coffee varieties. Among the several coffee polyphenols, chlorogenic acid is most affected by roasting levels [110]. Compared to Waialua coffee, Kaua’i coffee had higher concentrations of pyrogallol, which is known to inhibit cellular glutathione (GSH) and induce apoptosis (cell death) in human platelets [111].

Lipid content in coffee beans accounts for about 10–17% of their dry weight, and most of these lipids are triacylglycerols (TAGs) and small quantities of phospholipids (PLs). TAGs contain both saturated and unsaturated fatty acids (FAs), with unsaturated FAs being oleic (18:1(n-9)), linoleic (18:2(n-6)), and linolenic (18:3(n-3)) [112]. Coffee lipids are major contributors to organoleptic properties, quality, and formation and stabilization of coffee foam and emulsion and influence flavor and aroma, specifically in espresso coffees [113,114,115]. Lipids in coffee beans are influenced not only by growing conditions such as altitude, shade, and temperature [115,116,117], but also by genotype [113,116,117,118]. For example, Arabica coffees generally have lipid contents of 15%, while Robusta has about 10% lipids [113]. Palmitic (16:0), arachidic (20:0), stearic (18:0), and linolenic (18:3) acid contents are higher and oleic acid (18:1) content is lower in Arabica compared to Robusta [113,119,120]. Considering the fact that lipids play an important role in coffee bean development, coffee brew properties, and the effects of coffee on human health, few studies have focused on lipidomic profiles in coffee beans [112,113,121]). To our knowledge, our study is the first to investigate the effect of coffee varieties, roasting, and brewing on the lipidomic profiles of coffee brews. In our study, palmitic (16:0), stearic (18:0), and oleic (18:1) acids were higher in the Kaua’i compared to Waialua roasted coffee brews. No major differences in these FAs were noted in green coffee beans. Both green and roasted Waialua and Kaua’i coffee brews also differed in a few phospholipids (PC; 34:1, 36:2, 34:2), phosphatidyl ethanolamine (1-PE. 17:0/17:0), and several unannotated lipids that warrant further investigation into their identities.

Biogenic amines (BAs) in food and beverages are mainly formed due to the decarboxylation or amination of proteins and/or free amino acids via microbial or natural enzyme activity. Putrescine, spermidine, spermine, and serotonin are the most abundant BAs in coffee beans and coffee beverages, while cadaverine and tyramine are present in smaller amounts [122,123]. Processing methods of unripe coffee beans can affect the final levels of some BAs. Histamine, tryptamine, and cadaverine were detected in low-quality and defective coffee beans. The presence of BAs is an indicator of undesired microbial activity and at high concentrations can be toxic to humans [29,124,125]. BA concentrations have been studied to identify coffee origins, e.g., Asia, South America, and Africa [126].

Putrescine is the most abundant amine in both Robusta and Arabica coffee, followed by spermidine, spermine, and serotonin, while cadaverine and tyramine are generally present in smaller amounts [127,128,129,130]. Putrescine is used to discriminate coffee species, while tyramine is considered a chemical marker for *Angolan robusta*, and low levels of histamine are present in low-quality or immature coffee beans [129,131]. In our study, although brews of green beans or the roasted coffee of both varieties demonstrated differences in several biogenic amines, putrescine, spermidine, spermine, and serotonin, cadaverine and tyramine were not detected. The effect of roasting on BAs is still controversial since some studies report a reduction in BAs while other studies indicate high BA levels after roasting [29,122]. Overall, brewed coffee contains a very low level of BAs compared to green or roasted ground coffee beans [29].

Metabolites, lipids, and BA composition are also affected by methods of brewing coffee, as noted in 76 commercial coffees [36]. Studies have indicated that different brewing methods affect not only the chemical composition but also the aroma [36,63]. Metabolite variations are also influenced by temperature as in hot or cold brews [86], brewing time, or the size of ground solids [45,58]. In contrast to studies by Rothwell et al. [36], studies by Kim et al. [33] indicated that the composition of bioactive compounds was dependent upon roasting rather than brewing methods. Kim et al. [33] demonstrated significantly different levels of resveratrol, eugenol, ferulic acid, and vanillin between hot and cold brews, but only for dark roasted coffee. In our studies, we did not detect resveratrol, eugenol, or vanillin. However, ferulic acid was highest in our coffees brewed by filter mesh (hot brewing method) and French press (cold brewing temperatures) as compared to cold brew and filter paper (hot brew) methods. Differences between our study and that of Kim et al. [33] could be attributed to the type of coffee, roasting level, or temperatures of brewing. Similar to Kim et al.’s study [33], caffeine levels were unaffected by brewing methods in our study. Ferulic acid is known for its antioxidant, anti-inflammatory, and antimicrobial properties [33]. In our study, chlorogenic acid was the lowest in cold-brewed coffee compared to other brewing methods. Caffeine levels were unaffected by brewing methods, which is similar to the result noted by Kim et al. [33].

Among several biological properties, the health benefits of coffee are attributed to its antioxidant capacity [43,64,80,81,83,132,133,134,135,136]. A large variation in antioxidant capacity has been observed among commercially brewed coffees [135] as well as variety and origin of coffee [137], degree of roasting [138], type of roast and blend [139], and brewing methods [43,64,81,136,140]. Among the 12 varieties of Arabica and 1 variety of Robusta studied by Priftis et al. [132], roasting increased the antioxidant capacity in 1 coffee variety and reduced it in 5 varieties of Arabica and resulted in no difference in the other varieties. On the other hand, slower roasting speeds and dark roasts reduced antioxidant capacity, and lightly roasted coffee had more antioxidants [83,133]. Besides roasting conditions, the preparation of coffee brews with different coffeemakers also influenced their antioxidant capacity. Mocha coffee had the highest antioxidant capacity compared to filter, plunger, and espresso coffees [134]. Another study indicated that cappuccino, a milk-based coffee drink, had the highest antioxidant activity as compared to instant coffee and Turkish coffee [141]. Wolska et al. [80] further demonstrated that brewing methods did not affect the antioxidant capacity of Robusta coffee brews, but simple infusion had the highest activity in Arabica brews prepared by French press, espresso maker, overflow espresso, and Turkish coffee. In contrast to the results of Wolska et al. [80], antioxidant capacity was unaffected by coffee types in our study but was influenced by brewing methods. Similar to the study by Perez-Martinez et al. [134], we also demonstrate that filter coffee had lower antioxidant capacity compared to other brews.

Studies have also indicated that besides antioxidant capacity, brewing methods also influence the aroma and mineral contents of coffee [64,142]. To date, one study has demonstrated that among the five brews from a coffee shop in Szczecin, Poland, coffee prepared by espresso machine had the lowest antioxidant capacity, followed by French press < drip = simple infusion coffee < Aeropress [64]. Furthermore, coffees prepared by simple infusion and Aeropress had higher levels of magnesium, manganese, chromium, cobalt, and potassium, while the drip brew had higher silicon levels [64]. Similar to Janda et al. [64], our study also indicated that French press coffee had a lower antioxidant capacity. We also observed an effect of brewing methods on the mineral contents of the coffee. Although the results are incomparable between our study and that of Janda et al. [64] due to differences in brewing methods, the individual mineral values in French press-brewed coffees are different in the two studies possibly due to the coffee variety or degree of roasting.

## 5. Conclusions

Our studies demonstrate that levels of global metabolites, lipids, and biogenic amines were significantly influenced by roasting and differed between the two Hawai‘i-grown coffee varieties: “Kona Typica” and “Yellow Catuai”. Interestingly, the mineral contents and the antioxidant capacity of both these coffee varieties were influenced only by the brewing methods. Total minerals were higher in cold brews compared to other brewing methods. Similarly, regardless of the coffee variety, cold brew coffees had the highest antioxidant capacity, followed by coffees brewed by the French press method. Future studies are warranted to understand and extrapolate the influence of brewing methods on antioxidant capacity in vivo.

## Figures and Tables

**Table 1:** Mineral contents of Kaua’i and Waialua coffee prepared by different brewing methods. ^a,b,c^ Mean values with common letters do not differ (*p* < 0.05).

Mineral	Average RDA, AI, and UL in Males and Females in mg/day (19-70 years)	Minerals in 8 oz of Kaua’i Coffee (mg)				Minerals in 8 oz of Waialua Coffee (mg)			
		French Press (K-FrP)	Filter Paper (K-FP)	Filter Mesh (K-FM)	Cold Brew (K-CB)	French Press (W-FrP)	Filter Paper (W-FP)	Filter Mesh (W-FM)	Cold Brew (W-CB)
P (mg.day^−1^) ^1^	700 * 1250 **	5.721 ± 0.26 ^a^	3.352 ± 0.42 ^a^	4.524 ± 1.46 ^a^	12.128 ± 1.30 ^b^	5.511 ± 0.004 ^a^	4.315 ± 0.13 ^a^	4.572 ± 0.45 ^a^	14.074 ± 1.36 ^b^
K (mg.day^−1^) ^2^	2600 * 4700 **	110.395 ± 9.70 ^a,b^	79.83 ± 7.16 ^a^	108.60 ± 33.17 ^a,b^	137.26 ± 16.88 ^b^	112.26 ± 2.57 ^a,b^	101.65 ± 3.42 ^a,b^	112.74 ± 8.60 ^a,b^	171.61 ± 17.77 ^c^
Ca (mg.day^−1^) ^1^	1000 * 1300 **	2.883 ± 0.19 ^a^	2.116 ± 0.23 ^a^	2.363 ± 0.80 a	5.711 ± 0.56 ^b^	2.299 ± 0.10 ^a^	2.032 ± 0.18 ^a^	1.946 ± 0.12 ^a^	6.871 ± 0.36 c
Mg (mg.day^−1^) ^1^	310–320 * 400–420 *	11.17 ± 1.28 ^a^	7.31 ± 0.14 ^b^	8.69 ± 2.26 ^a,b^	16.78 ± 0.97 ^c^	8.92 ± 0.18 ^a,b^	7.45 ± 0.42 ^b^	7.562± 0.65 ^b^	18.61 ± 1.73 ^c^
Na (mg.day^−1^) ^2^	2300 *	7.00 ± 0.008 ^a^	5.384 ± 0.20 ^a^	5.977 ± 1.63 ^a^	8.773 ± 0.23 ^b,c^	6.654 ± 0.05 ^a,b^	5.92 ± 0.13 ^a^	6.309 ± 0.86 ^a^	9.99 ± 1.26 ^c^
Fe (mg.day^−1^) ^1^	18 * 8 *	0.0084 ± 0.0011 ^a,d^	0.0073 ± 0.0007 ^d^	0.01 ± 0.002 ^a,d^	0.017 ± 0.001 ^b^	0.011 ± 0.0008 ^a^	0.011 ± 0.00001 ^a,d^	0.012 ± 0.001 ^a^	0.029 ± 0.003 ^c^
Mn (mg.day^−1^) ^2^	2.3 **	0.056 ± 0.004 ^a^	0.036 ± 0.008 ^a^	0.05 ± 0.02 ^a^	0.156 ± 0.03 ^b^	0.057 ± 0.0004 ^a^	0.046 ± 0.003 ^a^	0.05 ± 0.003 ^a^	0.252 ± 0.009 ^c^
Zn (mg.day^−1^) ^1^	8 * 11 *	0.006 ± 0.0002 ^a^	0.004 ± 0.001 ^a^	0.006 ± 0.001 ^a^	0.014 ± 0.003 ^b^	0.006 ± 0.001 ^a^	0.004 ± 0.00002 ^a^	0.007 ± 0.003 ^a,b^	0.023 ± 0.006 ^c^
Cu (μg.day^−1^) ^1^	0.9 **	0.005 ± 0.0001 ^a^	0.004 ± 0.0004 ^a^	0.006 ± 0.001 ^a^	0.009 ± 0.001 ^a^	0.005 ± 0.0001 ^a^	0.006 ± 0.001 ^a^	0.006 ± 0.001 ^a^	0.034 ± 0.023 ^b^
B (mg.day^−1^) ^3^	1.5	0.042 ± 0.003 ^a,c^	0.035 ± 0.003 ^a,b^	0.048± 0.01 ^a.c^	0.025 ± 0.003 ^b^	0.044 ± 0.0008 ^a,c^	0.048 ± 0.003 ^a,c^	0.052± 0.003 ^c^	0.039 ± 0.004 ^a^

^1^ Recommended dietary allowance (RDA); ^2^ adequate intake (AI); ^3^ tolerable upper intake level (UL); K-, Kaua’i; W-, Waialua; FrP, French press; FP, filter paper; FM, filter mesh; CB, cold brew. Sourced from * the Dietary Guidelines for Americans 2020–2025, ** FDA.gov (accessed on 10 November 2022).

## Data Availability

All data supporting reported results can be found in this article and supplementary materials.

## Supplementary Materials

The supporting information can be downloaded at: https://www.mdpi.com/article/10.3390/metabo13030412/s1.
